# Safety evaluation and hypolipidemic ability of water-soluble blue pigment extracted by HPD-400 resin from *Quambalaria cyanescens*

**DOI:** 10.71150/jm.2412011

**Published:** 2025-11-30

**Authors:** Ruobing Shi, Chengzhong Wang, Nianping Xue, Zhiguo Zhang

**Affiliations:** School of Food Science and Engineering, Qilu University of Technology (Shandong Academy of Sciences), Jinan 250353, P. R. China

**Keywords:** *Quambalaria cyanescens*, blue pigment, toxicity, liver, kidney, natural product, hypolipidemic ability

## Abstract

The oral administration of synthetic drugs can effectively reduce blood lipid levels, but adverse reactions may occur. Because of this, the hypolipidemic ability of natural products has been increasingly investigated. We evaluate the safety and hypolipidemic characteristics of a water-soluble blue pigment extracted using HPD-400 resin from the fungus *Quambalaria cyanescens*. Hypolipidemic ability was examined by constructing a hyperlipidemia model with different doses of blue pigment (50, 100, and 200 mg/kg. mouse body weight) for 28 d. Blue pigment purity increased from 20.32% to 70.70% following treatment with HPD-400 resin. Acute toxicity tests revealed blue pigment sourced from *Q. cyanescens* to have no toxic effects on mouse body weight, mortality, or behavioral characteristics. Subacute toxicity tests revealed no significant differences in food intake, body weight, or organ weights between treatment groups and controls. Histopathological examination of the liver and kidney tissues of mice administered blue pigment were normal, and serum enzyme activities and blood constituents were also within normal ranges. Blue pigment can significantly reduce the weight of mice, reduce liver and kidney damage and fat accumulation. It can also reduce total cholesterol, triglyceride and low density lipoprotein cholesterol in serum and liver tissue, and increase the level of high density lipoprotein cholesterol. Reduce the levels of alanine aminotransferase, aspartate aminotransferase, alkaline phosphatase, creatinine, urea and uric acid in serum. Increase the activities of total superoxide dismutase, glutathione peroxidase and catalase in serum and liver tissue, reduce the content of malondialdehyde, and up-regulate liver lipase and lipoprotein lipase. Our work proves that blue pigment is nontoxic, has the function of reducing blood lipid, and can alleviate obesity-related symptoms by regulating lipid metabolism and oxidative stress.

## Introduction

As society ages and living standards improve, the prevalence of hyperlipidemia increases. Hyperlipidemia is characterized by dyslipidemia and lipid metabolism disorders, which also increase the risk of chronic diseases (Cheng et al., 2023; Tang et al., 2024; Touiss et al., 2017). While statins, fibrates, and nicotinic acids are often used in clinics to treat these disorders, their long-term use has deleterious side effects such as hepatotoxicity and insulin resistance (Neuvonen et al., 2006; Stroes et al., 2015). Therefore, alternative, safe and non-toxic natural lipid-lowering substances have been increasingly searched for and investigated.

Fungus can produce abundant secondary metabolites such as alkaloids, steroids, terpenoids, flavonoids, anthraquinone, and naphthoquinone. These compounds have different structures and various biological activities. For example, lovastatin is a well-known lipid-lowering drug that is mainly sourced from fungi such as *Penicillium citrinum*, *Monascus ruber*, and *Penicillium steckii* (Phainuphong et al., 2016; Srinivasan et al., 2022; Upendra et al., 2013; Yu et al., 2018). Therefore, fungi are potentially the source of lipid-lowering substances

The fungus *Quambalaria cyanescens* (Basidiomycota) can be screened from red rice, air, insect larvae, and grape leaves (Meshram et al., 2022; Narmani and Arzanlou, 2019; Stodůlková et al., 2008). The liquid fermentation of *Q. cyanescens* produces considerable water-soluble blue pigment that has rich biological activities (e.g., anti-oxidation, anti-aging, and hypoglycemic activities, in vitro) (Huang, 2023; Stodůlková et al., 2015; Zhu et al., 2023). However, there is no report to explore the potential of blue pigment in reducing blood lipid.

Safety evaluation of fungal pigments is necessary for application in the food industry, because fungi may produce toxins while producing pigments and other active substances, and toxins affect the application of fungal pigments in the food industry (Copetti, 2019). The monascus pigment and toxin-citrinin are produced by liquid fermentation of monascus, and institutions such as the European Union and the United States prohibit the application of monascus pigment in the food industry to ensure human health (Pandit et al., 2020; Poorniammal et al., 2021). Moreover, the toxicity data of the blue pigment from *Q. cyanescens* are still limited.

In previous studies, blue pigment was separated by membrane filtration, but this method was time-consuming and costly. At present, macroporous adsorption resin is widely used because of its good selectivity, high safety, reusability and expansion of production scale. In this study, blue pigment was separated and purified by macroporous adsorption resin (HPD-400) to further explore its safety and hypolipidemic activity. It is no toxicity food with lipid-lowering function. This study provides a large number of data basis for the following component analysis and lipid-lowering mechanism research of blue pigment.

## Materials and Methods

### Materials and instruments

*Quambalaria cyanescens* was screened from red yeast rice and maintained in our laboratory. The strain was activated every 2–3 months using a potato dextrose agar medium. HPD-400 resin was purchased from Zhengzhou Hecheng New Material Technology Co., Ltd. (China). Chemical reagents and organic solvents (analytical grade) were obtained from Sinopharm Chemical Reagent Co., Ltd., and reagent kits were purchased from Nanjing Jiancheng Biotechnology Co., Ltd.

Experimental equipment included a BXYC-WX2200 horizontal intelligent precision shaking table (Shanghai Boxun Medical Biological Instrument Co., Ltd.), SCIENTZ-10ND vacuum freeze-dryer (Ningbo Xinzhi Biotechnology Co., Ltd., China), Synergy neo2 multifunctional enzyme-labeled instrument (Berten Instrument Co., Ltd., USA), BC-5000 Vet automated hematology analyzer (Mindray Medical International Co., Ltd., China), 722 ultraviolet spectrophotometer (Shanghai Yidian Analytical Instrument Co., Ltd.), HistoCore BIOCUT Manual rotary microtome (Shanghai Leica instrument Co., Ltd.), and an E100 microscope (Nikon Co., Ltd., Japan).

### Blue pigment preparation

After liquid fermentation for 14 d (28°C), mycelia were removed by centrifugation. The supernatant was concentrated to 1/5 of its original volume and then treated with three times the volume of ethanol. The residue was removed by centrifugation, concentrated by spinning, and freeze-dried to obtain a crude blue power. HPD-400 resin was used to dynamically treat this powder by column chromatography. The optimal process included: an adsorption flow rate of 2.0 ml/min, sample concentration of 0.9 mg/ml, desorption solution of 86.85% ethanol, desorption flow rate of 1.49 ml/min, and elution volume of 8.86 BV. The blue pigment powder was obtained by freeze-drying.

### Determining blue pigment purity

Following Zhu et al. (2023), blue pigment purity was analyzed by HPLC (1260, Thermo Fisher Scientific, USA) and chromatographic column (InertSustain, AQ-C18, 5 μm, 4.6 × 250 mm). Mobile phases A and B involved 50 mmol/L potassium dihydrogen phosphate solution (pH 3.0) and 100% acetonitrile, respectively. The elution profile with time in min/% A was: 0 min - 90% A; 5 min - 90% A; 30 min - 45% A; 34 min - 45% A; 35 min - 90% A. Column temperature, flow rate and detection wavelength were set at 30°C, 1.0 ml/min, and 254 nm, respectively.

### Experimental animals

Animal experimentation proceeded in accordance with Shandong Normal University ethics approval (AEECSDNU2024003). Female rats (8 weeks old, n = 6) were used in the acute toxicity test, mice (4 weeks old, n = 40, 50% male) were used in a subacute toxicity test; and male mice (4 weeks old, n = 50) were used in the hypolipidemic test. Animals, having been fed a common high-fat diet (D12492), were purchased from the Beijing Weitong Lihua Experimental Animal Technology Co., Ltd. Animals were housed in polycarbonate cages with 40–60% relative humidity, 25 ± 3°C temperature, and a 12/12 light/dark cycle. Animals could feed and drink freely. Corncob padding was replaced every two days. Animals were acclimated for 7 days before experimentation.

### Acute toxicity study

The study followed Organization for Economic Cooperation and Development (OECD) guidelines, specifically acute toxicity classification method 423 (Jonsson et al., 2013). Female rats were divided into two groups, each containing three rats. Freeze-dried blue pigment dissolved in physiological saline was used in experiments. Control group rats were treated with normal saline solution, whereas treatment group rats were administered a blue-pigment dose of 50, 300, 1000, or 2000 mg/kg (body weight) orally. During administration, any signs of toxicity were observed to be delayed for 3 d. Rats were examined for 4 h prior to each dose administration, and afterwards every 12 h for 14 d. Rats were weighed daily, and mortality and behavioral characteristics were recorded. On completion of the experiment, rats were fasted overnight and euthanized with anesthetic (urethane, 1,500 mg/kg). Major organs (liver, lung, heart, spleen, and kidney) were removed, washed with normal saline, wiped dry, and weighed. Relative organ weight (organ weight/fasting weight) was calculated.

### Subacute toxicity study

**Experimental animal maintenance and diet:** According to the OECD guideline 407, we examined subacute toxicity to determine the safety of the *Q. cyanescens* blue pigment (Pandit et al., 2020). Mice were randomly divided into four groups, with five males and females in each. Freeze-dried blue pigment dissolved in physiological saline was used to feed animals. Blue pigment (250, 500, and 1,000 mg/kg mouse body weight) was given to the mice each day for four weeks; control-group mice received the same volume of normal saline. Mouse mortality and behavior were recorded daily. Daily food intake and weekly weight changes in mouse weight were recorded. The pigment dosage was adjusted according to the weight of mice every week. After experimentation, mice were fasted overnight. After euthanasia (cervical dislocation), the liver, lung, kidney, spleen, thymus, and gonad tissues were removed, washed with normal saline, wiped dry, and weighed. Relative organ weight was calculated. Blood was collected in single tubes with and without EDTA for hematology and biochemical analysis, respectively.

**Histopathology, hematology, and clinical chemistry:** The liver, kidney, and heart were fixed in 10% formaldehyde for 24 h, embedded in paraffin, and sectioned by microtome. Section thickness was 5 µm. Sections were then stained with hematoxylin and eosin, and tissue structure was observed by light microscopy.

An automated hematology analyzer was used to directly measure the concentration of blood components (hemoglobin concentration [Hb], hematocrit [PCV], red blood cells [RBC], white blood cells [WBC], average red blood cell hemoglobin [MCH], and average red blood cell hemoglobin concentration [MCHC]). Components were then compared to see whether they were within the normal range. Analyses were performed in parallel, three times.

Blood was left at 37°C for 1–2 h. Serum was collected after centrifugation (1006.20 g, 20 min). Aspartate aminotransferase (AST), alanine aminotransferase (ALT), alkaline phosphatase (ALP), lactate dehydrogenase (LDH), γ-glutamyl transferase (GGT), creatinine (CRE), urea (Urea), and total cholesterol (TC) contents in serum were calculated using diagnostic kits. Analyses were performed in parallel, three times.

### Hypolipidemic activity

**Experimental animal maintenance and diet:** Mice were randomly divided into five groups, with 10 individuals in each (Table 1). Body weight and food intake were recorded weekly, and pigment dosage was adjusted according to mouse body weight each week. The experiment ran for eight weeks.

**Collection of blood and organ tissues:** Mice were fasted overnight after experimentation, and blood was collected from their eyes. After euthanasia (cervical dislocation), the spleen, liver, kidney, heart, and perirenal and gonadal fat were quickly removed, washed with normal saline, and wiped dry. Organ weight was recorded. Relative organ weight was calculated. Blood was left at 37°C for 1–2 h, and serum was collected after centrifugation (1006.20 g, 20 min).

**Biochemical estimations:** To prepare a 10% liver tissue homogenate, a liver sample (1.0 g) was weighed and cut into pieces; normal saline (9 ml) was added, homogenized completely at 4°C by grinder, centrifuged (2263.95 g, 30 min), and the supernatant was collected for analysis. Serum TC, TG, HDL-C, and LDL-C levels were determined using kits. The atherosclerosis index (AI) was calculated using the formula (AI = (TC-HDL-C)/HDL-C). Serum parameters of liver and kidney function were measured (ALT, AST, ALP, CRE, BUN, and UA). Analyses were performed three times in parallel.

**Estimation of antioxidant ability and total lipase activity:** Antioxidant ability was assessed using kits by measuring malondialdehyde (MDA) content, and superoxide dismutase (SOD) and glutathione peroxidase (GSH-PX) activities in serum and liver tissues. Liver homogenate protein content was determined by bicinchoninic acid assay (BCA); activities of lipoprotein lipase (LPL) and hepatic lipase (HL) in the liver were determined by kit. Analyses were performed three times in parallel.

### Data analysis

IBM SPSS Statistics 26 and Origin 8.0 software were used to process data and for graphical analysis. Data are expressed as means ± standard deviations. One-way ANOVA was used to examine for significant differences between treatment groups at thresholds of *P* < 0.05 (significant) and *P* < 0.01 (highly significant).

## Results and Discussion

### Blue pigment preparation and purity determination

Conventional methods of pigment separation include solvent extraction (Dong et al., 2014), silica gel column chromatography (Yu et al., 2022), and high-pressure liquid chromatography (Finger et al., 2019). Problems with these methods include health and safety risks, damage to pigment structure, and their complicated operation (Li and Chase, 2010). Macroporous adsorption resin column chromatography is, however, reusable, highly selective and safe, and relatively simple to operate. It is widely used to enrich and purify various natural active components such as terpenoids, flavonoids, and glycosides, to decolorize proteoglycans, and in the treatment of sewage (Hou et al., 2019; Wei et al., 2015; Yang et al., 2012). For example, Chen et al. (2021) used CAD-40 resin to separate yellow and orange pigments from *Monascus* fermentation broth, with pigment separation increasing to > 80% following treatment. For this reason, we selected HPD-400 resin to separate the blue pigment from *Q. cyanescens*.

After fermentation broth was dynamically treated with HPD-400 resin, and the solution’s absorbance increased by 34.79 ± 0.21%, possibly because impurities (e.g., polysaccharides, inorganic salt, and protein) were removed and the blue pigment’s purity was improved (Wen et al., 2022).

**Fig. 1.**
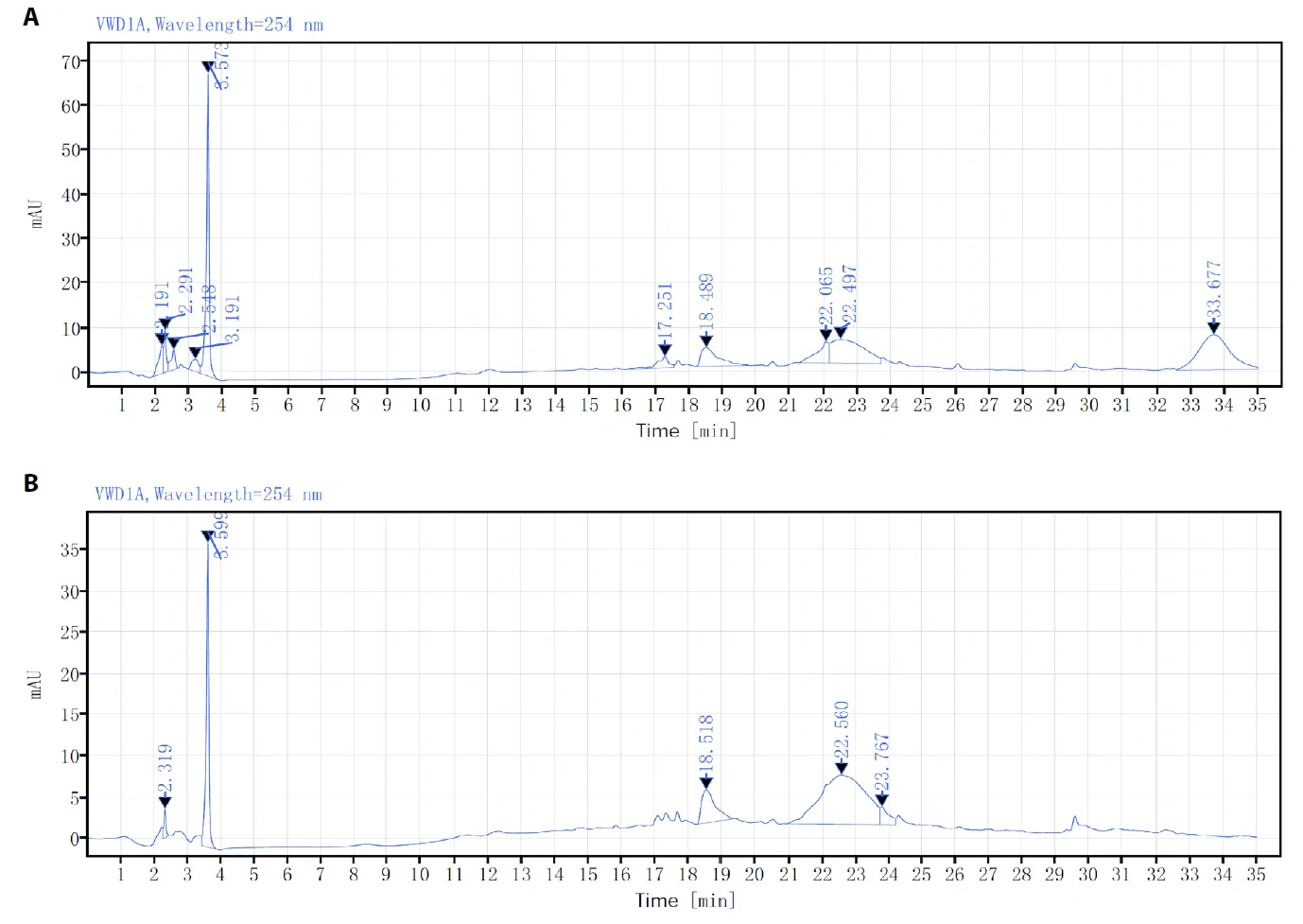
Blue pigment purity before (A) and after (B) treatment with HPD-400 resin by HPLC. Retention time: 22.497 (A), 22.560 (B).

Blue pigment powder obtained after microporous-adsorption resin treatment was a mixture (Fig. 1). But the specific composition is unknown, which has become the focus of later research. Blue pigment purity increased from 20.32% to 70.70% using HPD-400 resin, indicating that this resin treatment process could effectively purify the blue pigment target.

### Acute toxicity study

**Fig. 2.**
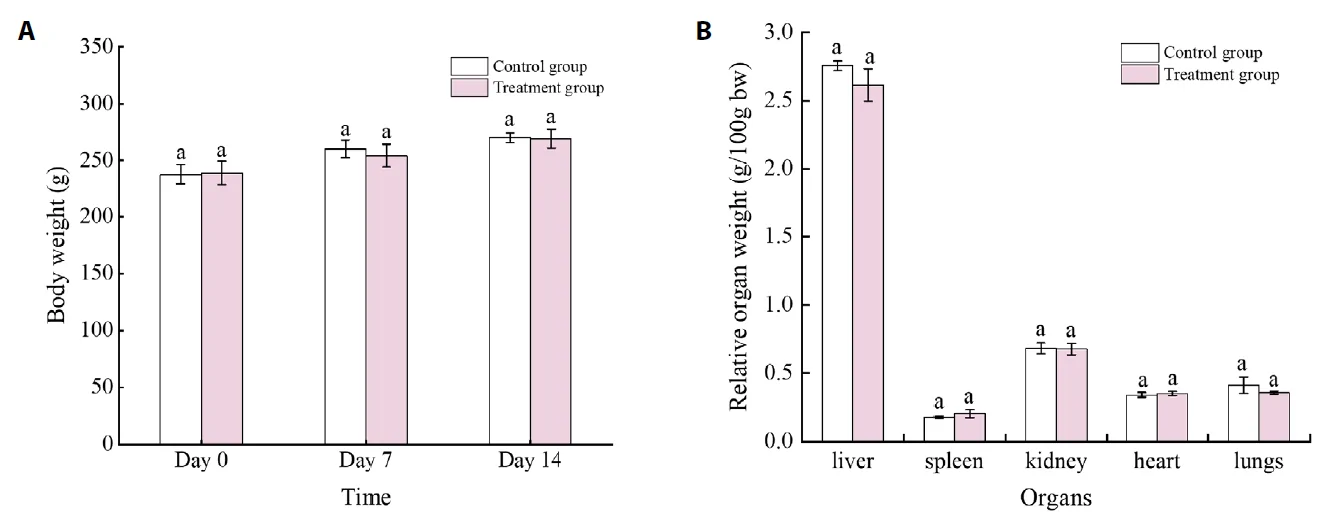
Effects of blue pigment on body weight (A) and relative organ weight (B) of female rats in an acute toxicity study. Data are expressed as means ± SD (n = 3 rats per group). Differences between treatment and control groups were not significant (*P* > 0.05).

No toxic clinical symptoms (e.g., coma, vomiting, death) occurred immediately or during post-treatment administration of blue pigment at 2,000 mg/kg mouse body weight. Compared with the control group, there were no significant differences in body weight (Fig. 2A) or relative organ weight (Fig. 2B) of the liver, spleen, kidney, heart, and lung tissues in pigment-treatment groups (*P* > 0.05). No treatment concentration of blue pigment was toxic and should be included in the fifth category. The LD50 for blue pigment exceeded 2,000 mg/kg (Fig. 2A) (Figueredo et al., 2018).

### Subacute toxicity study

**Fig. 3.**
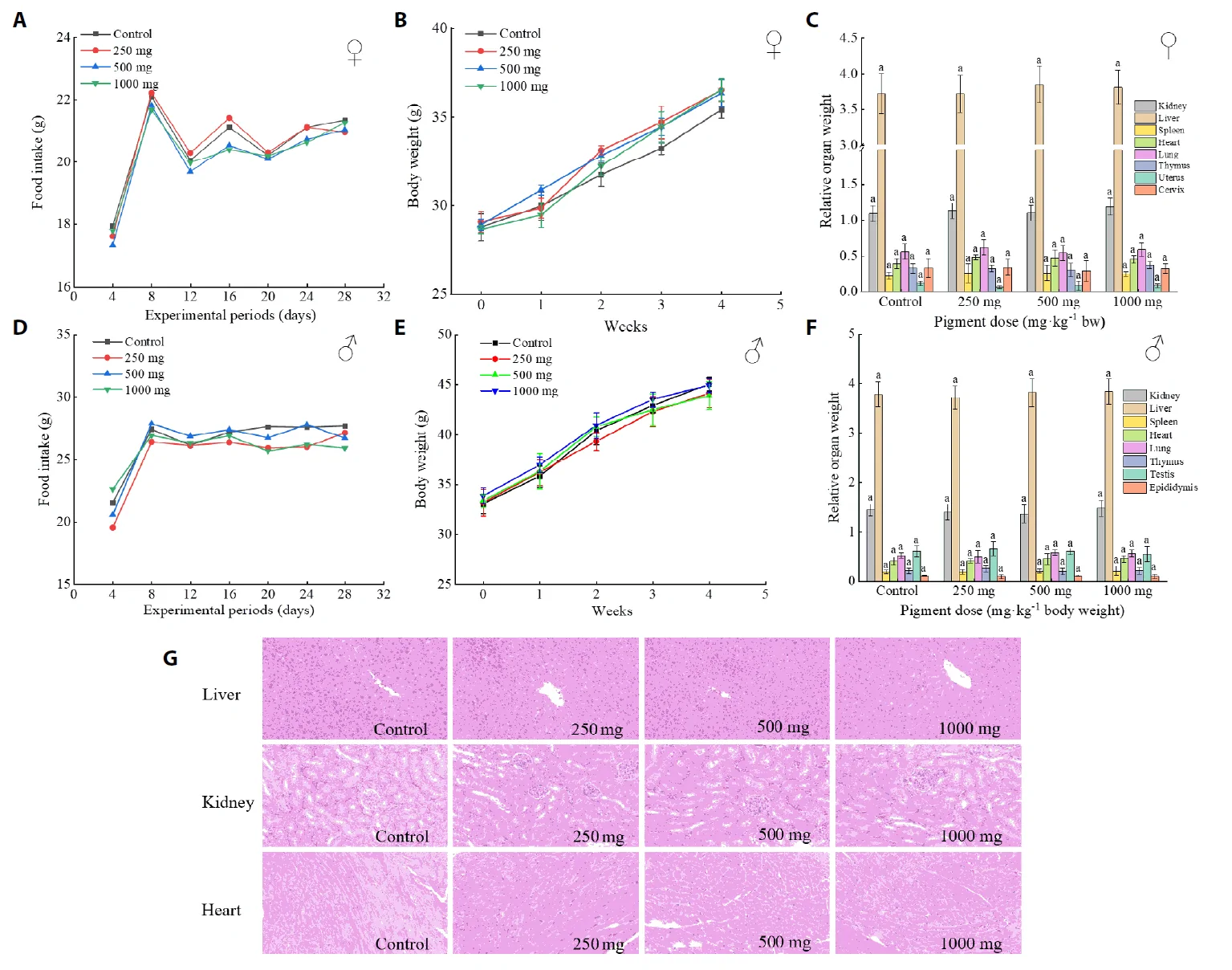
Effects of administering blue pigment after 28 d on food intake (A, D), body weight (B, E), relative organ weight (C, F), and organ morphology (G) in male and female mice in a subacute toxicity study. Magnification, 400×. Data are expressed as means ± SD (n = 5 mice per group). Differences between treated and control group mice were not significant (one-way ANOVA, *P* > 0.05).

**Food intake, and body and relative organ weights:** Food intake, and body and relative organ weights are important metrics of organ damage or abnormality (Amalraj et al., 2024; Pandit et al., 2020). There were no significant changes in food intake of male and female mice (*P* > 0.05) (Fig. 3A and 3D). Mouse weight gain and final weight also did not differ significantly between treatment- and control-group mice (*P* > 0.05) (Fig. 3B and 3E). At no treatment dosage were there any significant differences in liver, heart, spleen, kidney, lung, thymus, or gonad weights (male and female) (Fig. 3C and 3F). Accordingly, administering blue pigment at no treatment dose had any adverse effect on mouse food intake, body weight, or relative organ weight.

**Histopathology, hematology, and clinical chemistry:** Blood composition is important in toxicity studies (Pandit et al., 2020). Blood components after 28 d are detailed in Table 2. Hb, RBC, and WBC remained within normal ranges. Blue pigment did not adversely affect the blood of either male or female mice.

Serum enzyme activity is another important index in toxicity studies (Mukinda and Syce, 2007; Pandit et al., 2020). ALT, AST, GGT, LDH, ALP, CR, Urea, and TC are major parameters in liver and kidney tissues that indicate the health of these organs (Wang et al., 2022). Serum enzyme levels (Table 2) are all within normal ranges. Blue pigment had no toxic effect on serum enzyme activity.

Liver, kidney, and heart tissue structure were normal after 28 d (Fig. 3G). Liver tissue had normal hepatocytes and central veins; renal tissues had normal glomeruli, and proximal and distal convoluted tubules, with no pigment deposition; and the section of heart tissue was round or irregular, some cut to the nucleus, and normal perikaryon and lipofuscin particles were observed. Blue pigment did not adversely affect the structure of important organs in either male or female mice.

### Hypolipidemic activity

**Fig. 4.**
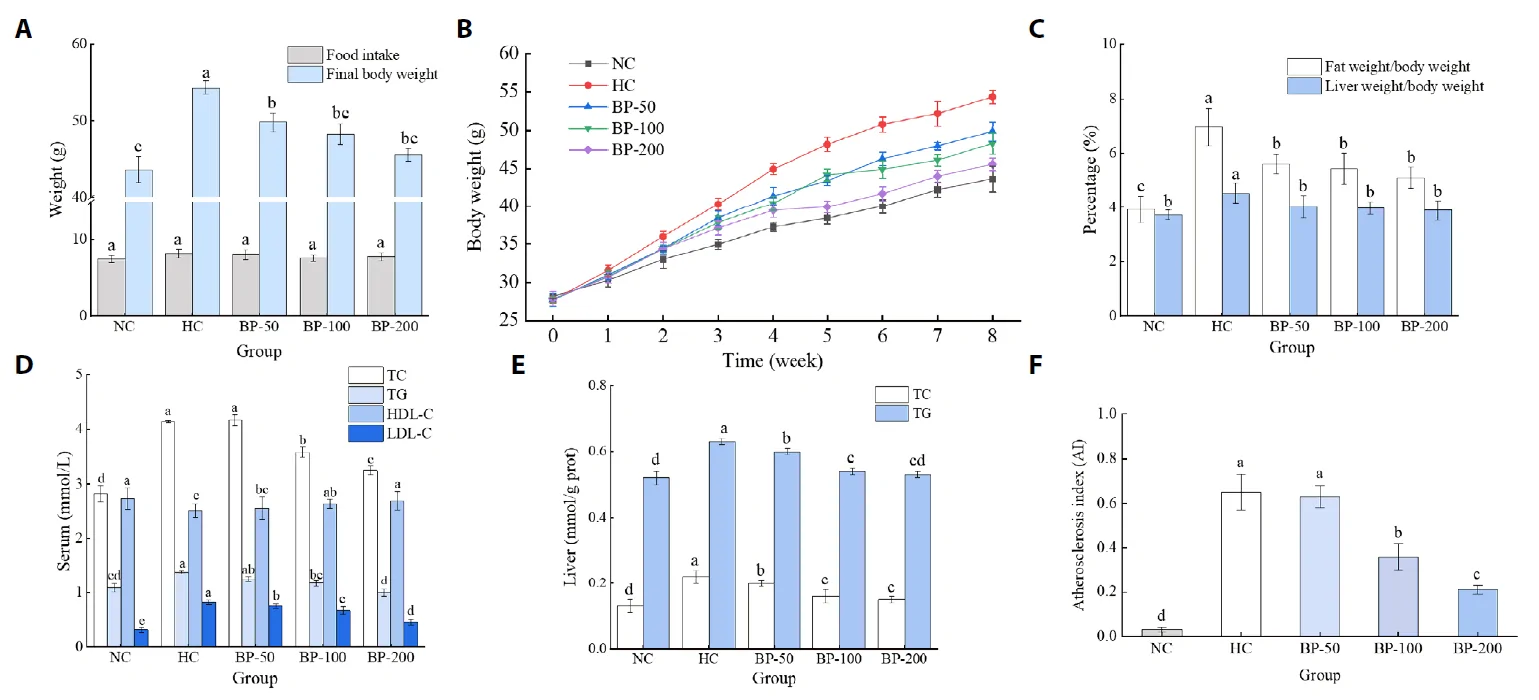
Effects on food intake (A), body weight (A, B), relative weight of fat (C), relative weight of liver (C), serum (D), and liver (E) lipid levels, and AI (F) in a hypolipidemic activity study. AI = (TC-HDL-C)/HDL-C. n = 10 mice per group. Different letters indicate significant differences between groups (one-way ANOVA, *P* < 0.05).

**Establishment of the hyperlipidemia model:** The effects of blue pigment on food intake, body weight, and relative organ weight in high-fat mice are illustrated in Fig. 4. Mice are considered obese if they gain > 20% of their initial body weight (Feng et al., 2024). Mice in the high-fat group gained > 20% of their initial body weight after 4 weeks on a high-fat diet (Fig. 4B). The weight in the high-fat group differed significantly from that in the NC group (*P* < 0.05), confirming that the high-fat model has been successfully established.

**Food intake, and body and relative organ weights:** The hypolipidemic effect may be related to several mechanisms, including reducing food intake, increasing bile acid excretion and reducing bile acid reabsorption, and producing short-chain fatty acids that may affect cholesterol synthesis and fat metabolism (Lin et al., 2024). There was no significant difference in food intake among mouse treatments, indicating that blue pigment did not reduce blood lipids by reducing appetite or food intake (*P* > 0.05) (Fig. 4A).

Obesity is an independent risk factor for hyperlipidemia (Yu et al., 2018). The body weight (Fig. 4A and 4B), relative weight of fat (Fig. 4C), and liver relative weight (Fig. 4C) in the HC group increased significantly by 24.52%, 77.55%, and 14.02% compared with the NC treatment, respectively (*P* < 0.05), possibly because a long-term high-fat diet led to obesity and fat accumulation. Body weight, liver relative weight, and fat relative weight in the pigment-given groups were significantly lower than in the HC group (*P* < 0.05) and almost the same as levels in the NC group. Body weight, relative weight of fat, and relative weight of liver in the BP-200 group decreased significantly by 12.48%, 26.58%, and 8.04%, respectively (*P* < 0.05). Blue pigment effectively reduced body weight, and fat and liver weight in hyperlipidemic mice.

**Serum and liver lipid levels:** The liver plays an important role in fat metabolism, including fatty acid and triglyceride synthesis and storage, bile secretion and excretion, lipolysis, and cholesterol metabolism (Wang et al., 2022). The increase in TC, TG, LDL-C, and AI, and the decrease in HDL-C may lead to obesity, diabetes, hyperlipidemia, and atherosclerosis, and cause serious diseases of the heart and nervous system (Tang et al., 2023).

Effects of blue pigment on serum and liver lipid levels in high-fat mice are illustrated in Fig. 3D. Mice in the HC group had significantly higher TC and TG serum and liver levels, increased serum LDL-C levels and AI, and lower serum HDL-C levels compared with NC group mice (*P* < 0.05) (Fig. 4D–4F), suggesting that a high-fat diet led to dyslipidemia and atherosclerosis. Compared with the HC group, TC and TG levels in serum and liver tissues, and LDL-C and AI levels in serum were significantly lower, and HDL-C levels in serum were significantly higher in pigment-given mice (*P* < 0.05). Levels of TC, TG, LDL-C, and AI were significantly lower by 21.50%, 27.54%, 45.12%, and 67.69% in the BP-200 group, respectively; HDL-C levels were significantly higher by 7.17%; and TC and TG levels in the liver were significantly lower by 31.82% and 15.87%, respectively. These results demonstrate that blue pigment improved lipid levels in the serum and liver of mice and inhibited atherosclerosis. The effect was best in the BP-200 group. Mouse weight loss in pigment-given groups may be related to improved lipid levels.

**Antioxidant ability:** Oxidative stress is a potential factor associated with dyslipidemia and various metabolic disorders. Reactive oxygen species are closely related to lipid peroxidation (Harman, 1980). SOD and GSH-PX are essential antioxidant enzymes. MDA is an important indicator of anti-lipid peroxidation because it is the main oxidant of polyunsaturated fatty acids (Incalza et al., 2018).

**Fig. 5.**
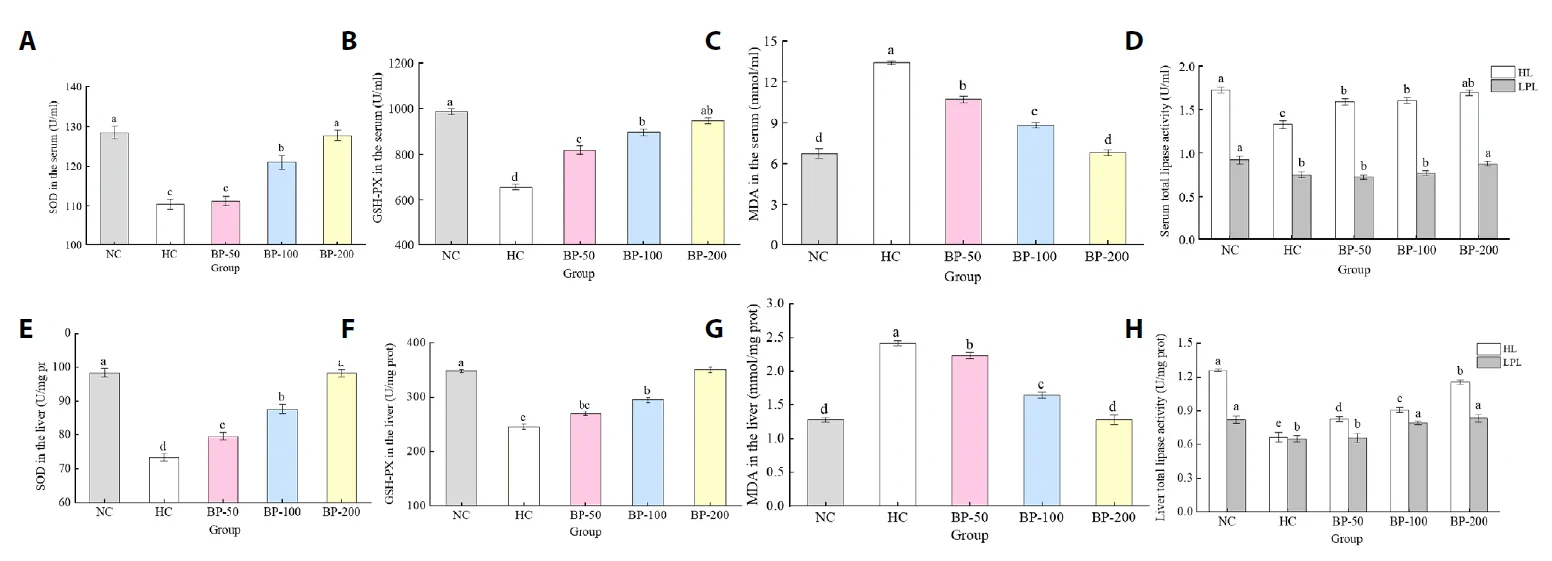
Effects of administering blue pigment on serum and liver SOD (A), GSH-PX (B), MDA (C), and total lipase levels (D) in a hypolipidemic activity study. Different letters indicate significant differences between groups (one-way ANOVA, *P* < 0.05).

The effects of administering blue pigment to mouse serum and liver antioxidant indices are illustrated in Fig. 5. MDA (Fig. 5C and 5G) was significantly higher and SOD (Fig. 5A and 5E) and GSH-PX (Fig. 5B and 5F) were significantly lower in the serum and liver in HC group mice compared with NC group mice (*P* < 0.05). This indicates that a high-fat diet induced oxidative stress and lipid peroxidation in liver tissue in vivo, reducing antioxidant capacity. MDA in serum and liver tissues of high-fat mice treated with blue pigment decreased significantly, while SOD and GSH-PX activities increased significantly compared with HC group mice (*P* < 0.05); a concentration-dependent relationship was apparent. These results are consistent with previous findings that blue pigment has excellent antioxidant activity in vitro (Pandit et al., 2020). The hypolipidemic mechanism of blue pigment from *Q. cyanescens* might be associated with antioxidant capacity. Enhanced antioxidant capacity might inhibit lipid peroxidation and play a role in hypolipidemia.

**Total lipase activity:** The effect of blue pigment on total lipase activity in the serum of mice is illustrated in Fig. 5. LPL and HL decompose TG and hydrolyze it into glycerol and free fatty acids, which participate in regulating lipoprotein metabolism (Yang et al., 2006). Levels of HL and LPL in serum and the liver in HC group mice were significantly lower than those in NC group mice (*P* < 0.05) (Fig. 5D and 5H). Activities of LPL and HL in the liver of mice in the BP-100 and BP-200 treatments were significantly higher than those in the HC group (*P* < 0.05). Activities of HL and LPL in the serum of the BP-200 treatment mice increased significantly by 27.32% and 16.81%, respectively (*P* < 0.01). These results indicate that blue pigment reduced blood lipids by increasing total lipase activity in the serum and liver tissues.

**Fig. 6.**
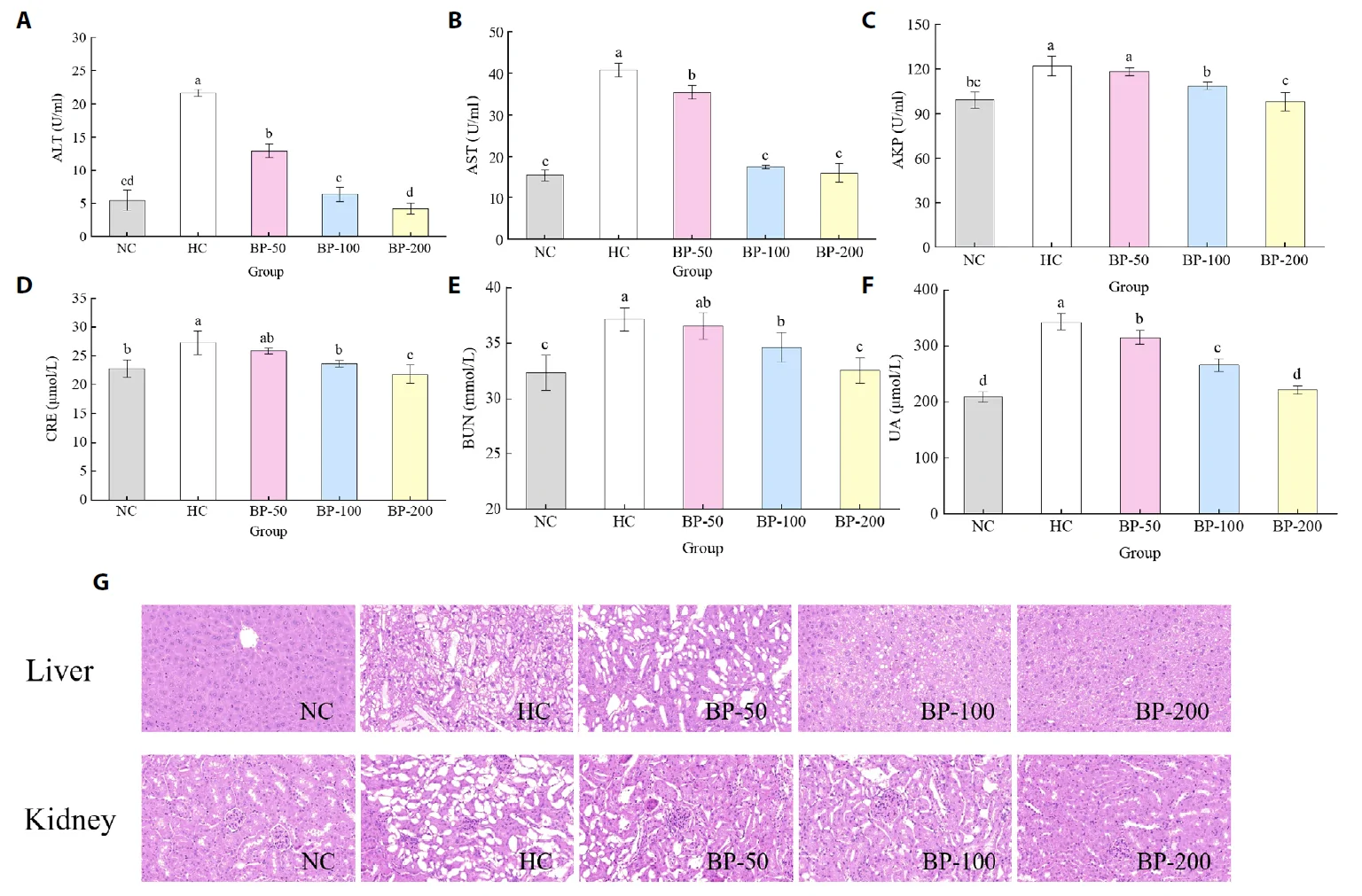
Effects of administering blue pigment on AST (A), ALT (B), AKP (C), CRE (D), BUN (E), UA (F), and liver and kidney tissue structure (G). Different letters indicate significant differences between groups (one-way ANOVA, *P* < 0.05).

**Liver and kidney function parameters:** AST, ALT, and AKP can indicate the degree of liver damage (Lin et al., 2024). CRE, BUN, and UA are the main indexes used to evaluate renal function, and an increase in them indicates damage to glomerular filtration function (Wang et al., 2022). The effect of blue pigment on liver and kidney function parameters of high-fat mice is illustrated in Fig. 6. Compared with the NC group, the activities of AST (Fig. 6A), ALT (Fig. 6B), and AKP (Fig. 6C) increased significantly in mice in the HC group (*P* < 0.01). We speculate that a high-fat diet caused liver damage. Activities of AST, ALT, and AKP in pigment-given mice were significantly lower than those in the HC group (*P* < 0.01). Among them, the BP-200 group had the best effect, because AST, ALT, and AKP decreased by 60.70%, 80.73%, and 19.68%, respectively. The contents of CRE (Fig. 6D), BUN (Fig. 6E), and UA (Fig. 6F) in the HC group increased significantly compared with the NC group (*P* < 0.01). We speculate that a high-fat diet also caused kidney damage in mice. Compared with the HC group, the serum levels of CRE and BUN were significantly lower in BP-100 and BP-200 group mice (*P* < 0.05). Serum levels of UA were reduced considerably in all pigment-given groups (*P* < 0.05). In summary, the blue pigment reduced related functional parameters of serum liver and kidney in high-fat mice in a concentration-dependent manner. Blue pigment might protect liver and kidney tissues by restoring serum enzyme levels, thereby reducing blood lipid levels.

**Histopathology:** The liver and kidney play important roles in the body. The hypolipidemic effect of blue pigment was evaluated by analyzing liver and kidney tissue sections. The effects of blue pigment on liver and kidney structure in high-fat mice are depicted in Fig. 6G. Mice in the NC group had structurally intact livers, with blue, rounded and centrally located nuclei in cells; no fatty deposits were observed. There was slight evidence of necrosis and fat infiltration in the HC group, and numerous lipid droplets and vacuoles were observed in the cytoplasm of hepatocytes and kidneys, with displacement of the nucleus to one side. These findings indicate that a high-fat diet might have caused organ damage. Compared with the HC group, the lipid droplets decreased in the liver and kidney tissue sections of mice in pigment-treatments. Additionally, fatty degeneration decreased and there was some recovery in cell morphology. The effect of blue pigment on improving tissue structure depended on its concentration. Results demonstrate that the blue pigment of *Q. cyanescens* can effectively improve the liver and kidney structure in mice fed a high-fat diet.

## Conclusion

We report on the acute and subacute toxicity, and anti-hyperlipidemia and antioxidant activity of blue pigment sourced from *Q. cyanescens* on rats and mice, in vivo. Different concentrations of blue pigment had no toxic effects on weight, hematological and biochemical parameters, or tissue structure of experimental animals. Blue pigment improved the weight, lipid level, serum enzyme activity, and tissue structure of mice with hyperlipidemia and enhanced the antioxidant capacity in vivo. These results demonstrate that blue pigment, a natural product, is safe to administer to rodents, and that it has high hypolipidemic activity. However, the structure of the blue pigment must be determined, and further analysis of how it lowers blood lipids is required to verify its application in food and other industries.

## Figures and Tables

**Table 1.** Animal grouping and handling procedures for hypolipidemic activity

Number	Group	Treatment
1	Blank control group (NC)	Normal diet + normal saline
2	High-fat model control group (HC)	High-fat diet + normal saline
3	Low-dose group (BP-50)	High-fat diet + 50 mg/kg/d BP
4	Medium-dose group (BP-100)	High-fat diet + 100 mg/kg/d BP
5	High-dose group (BP-200)	High-fat diet + 200 mg/kg/d BP

n = 10 male rats per group. BP, blue pigment

**Table 2.** Effect of blue pigment after 28 d on blood components and clinical biochemistry profile of male and female mice (subacute toxicity study)

Parameters	Daily dose (28 Days)							
	Control		Group Ⅰ (250 mg/kg bw)		Group Ⅱ (500 mg/kg bw)		Group Ⅲ (500 mg/kg bw)	
	Male	Female	Male	Female	Male	Female	Male	Female
Hb (g/L)	142.12 ± 12.73^a^	142.45 ± 15.56^a^	140.56 ± 14.85^a^	142.43 ± 11.88^a^	143.27 ± 18.21^a^	140.53 ± 19.19^a^	141.51 ± 14.95^a^	141.94 ± 16.97^a^
RBC (10^12^/L)	8.68 ± 1.10^a^	8.63 ± 1.25^a^	8.63 ± 1.52^a^	8.46 ± 1.67^a^	8.69 ± 1.27^a^	8.40 ± 1.56^a^	8.93 ± 1.67^a^	8.76 ± 1.21^a^
WBC (10^9^/L)	3.31 ± 0.23^a^	3.40 ± 0.16^a^	3.67 ± 0.29^a^	3.55 ± 0.46^a^	3.34 ± 0.17^a^	3.56 ± 0.25^a^	3.32 ± 0.29^a^	3.47 ± 0.23^a^
PCV (%)	49.85 ± 2.19^a^	49.65 ± 2.02^a^	49.55 ± 2.86^a^	49.35 ± 2.19^a^	49.95 ± 3.05^a^	49.70 ± 2.83^a^	49.25 ± 3.14^a^	49.00 ± 2.13^a^
MCV (fL)	57.45 ± 1.91^a^	57.40 ± 0.71^a^	57.65 ± 2.19^a^	57.65 ± 2.05^a^	57.25 ± 2.32^a^	57.39 ± 2.16^a^	57.77 ± 1.09^a^	57.23 ± 1.77^a^
MCH (pg)	16.02 ± 0.84^a^	16.15 ± 1.48^a^	16.11 ± 0.18^a^	16.14 ± 0.50^a^	16.19 ± 0.74^a^	16.09 ± 1.41^a^	16.06 ± 0.81^a^	15.90 ± 0.57^a^
PLT (10^9^/L)	556.56 ± 68.49^a^	553.55 ± 64.35^a^	554.59 ± 71.22^a^	554.00 ± 65.46^a^	555.54 ± 65.76^a^	556.13 ± 69.60^a^	557.02 ± 70.31^a^	559.03 ± 74.95^a^
AKP (U/L)	123.87 ± 3.80^a^	121.95 ± 2.98^a^	124.03 ± 2.76^a^	121.89 ± 3.64^a^	123.91 ± 2.85^a^	121.80 ± 2.13^a^	123.84 ± 3.79^a^	121.88 ± 2.84^a^
AST (U/L)	143.89 ± 4.09^a^	145.72 ± 3.84^a^	142.86 ± 3.61^a^	144.77 ± 3.96^a^	143.82 ± 3.41^a^	145.18 ± 4.23^a^	143.92 ± 3.06^a^	145.66 ± 3.50^a^
ALT (U/L)	18.38 ± 1.47^a^	20.33 ± 2.39^a^	18.42 ± 2.33^a^	20.36 ± 2.43^a^	18.39 ± 2.28^a^	20.35 ± 1.37^a^	18.37 ± 1.42^a^	20.35 ± 2.32^a^
LDH (U/L)	967.89 ± 20.74^a^	972.72 ± 19.99^a^	969.24 ± 21.43^a^	965.96 ± 20.12^a^	980.48 ± 25.79^a^	968.65 ± 23.08^a^	967.17 ± 21.37^a^	976.52 ± 24.01^a^
GGT (U/L)	4.28 ± 2.16^a^	4.31 ± 3.09^a^	4.10 ± 2.83^a^	4.44 ± 2.85^a^	4.51 ± 1.98^a^	4.37 ± 2.38^a^	4.42 ± 2.12^a^	4.59 ± 2.77^a^
CRE (mg/dL)	14.67 ± 1.23^a^	15.03 ± 0.88^a^	15.83 ± 1.09^a^	15.72 ± 1.38^a^	16.42 ± 1.41^a^	15.62 ± 0.74^a^	14.34 ± 1.17^a^	16.10 ± 1.23^a^
Urea (mg/dL)	11.58 ± 0.83^a^	9.64 ± 1.23^a^	11.03 ± 0.75^a^	10.20 ± 1.04^a^	11.77 ± 0.82^a^	10.48 ± 0.31^a^	12.23 ± 0.64^a^	9.92 ± 0.81^a^
TC (mg/dL)	2.88 ± 0.27^a^	2.44 ± 0.48^a^	2.66 ± 0.65^a^	2.51 ± 1.09^a^	2.74 ± 0.38^a^	2.55 ± 0.09^a^	2.72 ± 0.53^a^	2.48 ± 0.73^a^

Data are expressed as Means ± SD (n = 5). No significant difference was found between groups (one-way ANOVA, *P* > 0.05). AKP, alkaline phosphatase; ALT, alanine aminotransferase; AST, aspartate aminotransferase; CRE, creatinine; GGT, gamma-glutamyl transferase; Hb, hemoglobin; LDH, lactate dehydrogenase; MCH, mean cell corpuscular hemoglobin concentration; MCV, mean corpuscular volume; PCV, packed cell volume; PLT, platelet; RBC, red blood cells; TC, cholesterol; WBC, white blood cells
